# Bond Behavior of FRP Bars in Lightweight SCC under Direct Pull-Out Conditions: Experimental and Numerical Investigation

**DOI:** 10.3390/ma15103555

**Published:** 2022-05-16

**Authors:** Mohammed A. Abed, Zaher Alkurdi, Jan Fořt, Robert Černý, Sandor Solyom

**Affiliations:** 1 John A. Reif, Jr. Department of Civil and Environmental Engineering, New Jersey Institute of Technology, Newark, NJ 07102, USA; 2 Department of Structural Engineering, Budapest University of Technology and Economics, 1111 Budapest, Hungary; zaherkurdi@edu.bme.hu; 3 Department of Materials Engineering and Chemistry, Faculty of Civil Engineering, Czech Technical University in Prague, Thákurova 7, 16629 Prague, Czech Republic; cernyr@fsv.cvut.cz; 4 Department of Construction Materials and Technologies, Budapest University of Technology and Economics, 1111 Budapest, Hungary; solyom.sandor@emk.bme.edu

**Keywords:** lightweight aggregate concrete, fiber-reinforced polymer, FRP, bond, pull-out test, ATENA 3D software

## Abstract

In recent decades, lightweight aggregate concrete (LWC) became a popular building material due to its desired properties. However, various attributes of LWC, such as bond behavior of used reinforcing, have not been described thoroughly. In this regard, LWC produced with 0%, 50%, and 100% expanded clay aggregate was designed, and the physical–mechanical properties were assessed for material characterization. Subsequently, the bond behaviors of LWC reinforced with steel, glass fiber reinforced polymer (GFRP), and basalt fiber reinforced polymer (BFRP) bars were evaluated by pull-out tests. The results of the experimental program allowed the effects of expanded clay aggregate incorporation on LWC properties to be quantified. The bond strength of BFRP bars was not affected by the replacement of coarse aggregate by expanded clay aggregate, whilst the GFRP bars showed lower bond strength values of LWC specimens. Contrarily, in the case of steel bars, both the bond strength and bond stiffness were higher for LWC specimens than for those of normal concrete. Finite element software ATENA 3D was used for simulation of the bond behavior of LWC, and the model validated by the experimental results referred to reasonably corresponding outputs.

## 1. Introduction

Rapid urbanization is accompanied by advanced requirements of building materials to provide sufficient strength and durability parameters, prolong the service life, and reduce the environmental impact [1]. In this regard, concrete is deemed as an abundant, available, and flexible building material that meets most of the requirements of an ideal building material [2]. Although concrete has several benefits, some barriers limit its use and thus create pressure to modify its composition. Specifically, self-compacting concrete (SCC) has attracted substantial attention within recent decades due to the fact that it is not necessary to compact it in any way due to its excellent workability performance. Other types of concrete improvement mainly consist of the utilization of reinforcing fibers and bars to increase the bending and tensile strength, viewing plain concrete as a brittle material [3,4,5].

The bond between the reinforcement and concrete matrix represents an important parameter that influences the applicability of such modified material for structural constructions. Bond strength is the resistance of separation between the reinforcing bar and the surrounding concrete, and it is built through adhesion, friction, and mechanical interlock between the reinforcing bar and the surrounding concrete [6,7]. The pull-out (P-O) test is usually used to measure the local bond strength and provide details about the bond behavior between concrete and reinforcing bars [8,9,10]. As described by Mousavi et al. [11], conventional concrete compacted by vibration suffers from bleeding and segregation that often result in deterioration of bonds between the reinforcement and the concrete matrix. The utilization of superplasticizers in SCC together with a fine fraction of aggregates improves the reinforcement–concrete bonds due to effective coverage of the reinforcement surface.

As follows from the above-mentioned facts, the strength of the bond between the reinforcement used, and the material matrix depends on the quality of the formed interfacial transition zone (ITZ) [12]. As reported by Castel et al. [13], a denser and wider ITZ can be observed in SCC mixes compared to conventional concrete.

The bond behavior of lightweight concrete (LWC) was studied by Trad et al. [14], who concluded that the bond strength is also affected by the strength of concrete. In the case of 40 MPa grade strength concrete, the strength of normal weight concrete showed higher bond strength than LWC; yet this was not the case when the strength of concrete was greater than 40 MPa. Lightweight aggregate (LWA) negatively affects the fresh properties of concrete due to its high porosity compared to normal weight aggregate. Among others, the water to cement (w/c) ratio has a significant influence on the bond strength of LWC, as a higher bond strength of LWC is observed when it is produced with a low w/c ratio [15]. The low w/c ratio in LWC provides better adhesion components compared to normal weight concrete.

At present, the issues accompanied with steel reinforcement in normal as well as SCC concrete are well described in the literature [16,17,18]. However, facing new challenges, steel bars have been replaced in order to reduce the issues associated with corrosion and consequent strength loss, and the performance of the bond between the selected bar type needs to be investigated [19]. In this regard, Han et al. [20] studied hybrid steel and fiber-reinforced polymer (FRP) reinforcement bars and revealed that utilization of hybrid reinforcement struggles with non-uniform strain distribution and excessive deflection. As reported, understanding the bond responses of various types of fibers in normal concrete has been of interest to many researchers who have studied various alternatives to steel reinforcement [21,22]. However, the variety of materials represents a robust task that needs to be resolved properly to provide reliable guidelines for efficient material design. In addition to the employed experimental techniques, computation modeling is viewed as a valuable tool for the prediction of material response and overall performance [23].

Nowadays, numerical modeling is a commonly used technique because experimental investigations are sometimes impossible, costly, or time-consuming [24]. Many researchers have used finite element (FE) software such as ABAQUS [25] and ATENA 3D [26] to gain a better understanding of the bonding mechanism and the effect of various elements on the bond behavior, as well as providing a complete explanation of the failure mode. Yu and Jeong [27] developed a model for studying the bond between different types of wire and concrete using ABAQUS software and indicated that the simulation results agree reasonably well with the test data. Tavares et al. [28] studied bond behaviors of different bar diameters using ATENA 3D software, and they showed numerical results in the range of experimental results with slight differences. Cheung et al. [29] developed an FE simulation of P-O tests to study the bond behavior between steel bars and high strength fiber reinforced cementitious composites using ATENA 3D software. They mentioned that the numerical results were in very good agreement with the experimental results.

This study provides a better understanding of the bond behavior of glass and basalt FRP bars in LWC as potentially more sustainable alternatives to traditional steel-reinforced concrete structures, offering several important benefits. Experimental and numerical evaluations were conducted to emphasize the importance of developing numerical models that help simulate and understand LWC behavior with fewer experiments required.

## 2. Materials and Methods

### 2.1. Materials

To achieve the objective of the current study, three SCC mixtures were produced, the main difference between the mixtures being the proportion of the LWA. The mixtures were given the following symbols: LW0%, LW50%, and LW100%. LW0% refers to SCC produced with only normal weight aggregate, LW50% refers to SCC produced by substituting 50% of the normal weight aggregate by LWA, while LW100% refers to SCC produced with only LWA. The substitution ratio of normal weight aggregate was considered by volume, and all mixing proportions are presented in Table 1.

**Figure 1:**
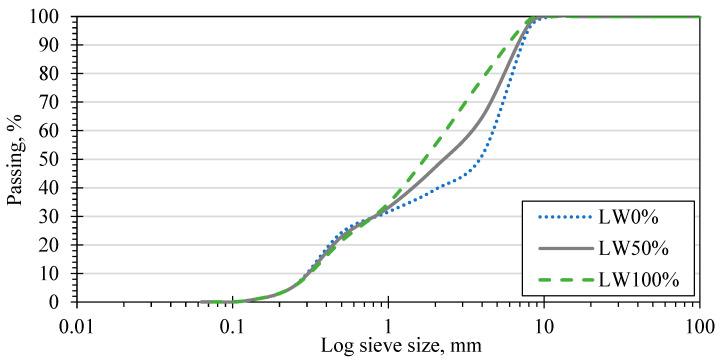
Sieve analysis for produced aggregate blends.

The primary materials used for SCC production were cement, fine aggregate, coarse aggregate, water, and chemical admixtures. The cement was Ordinary Portland Cement (OPC), while the fine aggregate was a local Hungarian natural quartz river (Danube River) fine aggregate. Two types of coarse aggregates with maximum sizes of 8 mm were used: natural aggregate as normal-weight aggregate, and expanded clay as LWA. For the total amount of aggregate, fine aggregate and coarse aggregate were used with proportions of 45% and 55%, respectively. Expanded clay was used with different proportions (0%, 50%, and 100%) of the normal weight aggregate mass. Sieve analyses for the three blends of aggregate are shown in Figure 1, while the physical properties of expanded clay are presented in Table 2.

**Figure 2:**
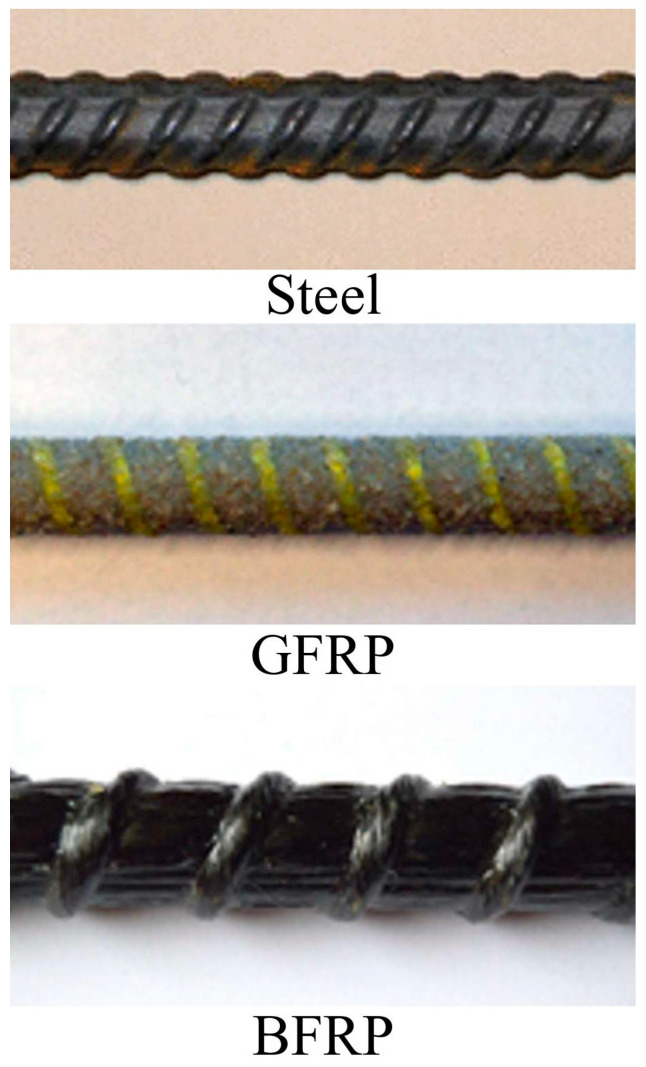
BFRP, GFRP, and steel bars.

To achieve the required strength and workability, as well as to satisfy the European guidelines for SCC [30], both BASF Glenium C300 and BASF Glenium 51 were used. Sand-coated glass fiber-reinforced polymer (GFRP), basalt fiber-reinforced polymer (BFRP), and steel B500B bars with diameters of 10, 14, and 8 mm, respectively, were used in the tested P-O specimens. The main properties of the reinforcements used are shown in Table 3, while the bars are shown in Figure 2.

### 2.2. Experimental Methods

The produced SCC mixtures were tested for their fresh properties, mechanical behavior, and P-O test. Proper fresh properties of SCC according to the European guideline for SCC [30] are ensured by the testing of slump flow and V-funnel methods. Table 4 presents the classes of consistency and viscosity expressed by slump flow and V-funnel time, respectively, as specified in the European guidelines for SCC.

Compressive strength, flexural strength, and splitting tensile strength were evaluated in accordance with European standards [31,32,33]. Shear strength was also tested using a novel experimental method consisting of a notched cylindrical push-off specimen creating two stress-free zones; this new system was proposed in another research paper [34]. The effect of LWA on the physical properties of concrete was evaluated by using a non-destructive technique (N-type Schmidt hammer). Cubic 150 × 150 × 150 mm specimens were used for compressive strength tests, 70 × 70 × 250 mm prisms for flexural strength tests, and Ø150 × 300 mm cylinders for the splitting tensile strength and shear strength tests. For every single property, three nominally identical specimens were tested, and the average was presented.

All specimens were kept in their molds for 24 h at a temperature of 22 ± 1 °C, after which they were de-molded and immersed in water to the age of 7 days and then kept under laboratory conditions at 20 ± 2 °C and 35% relative humidity until the testing date at the age of 28 days.

**Figure 3:**
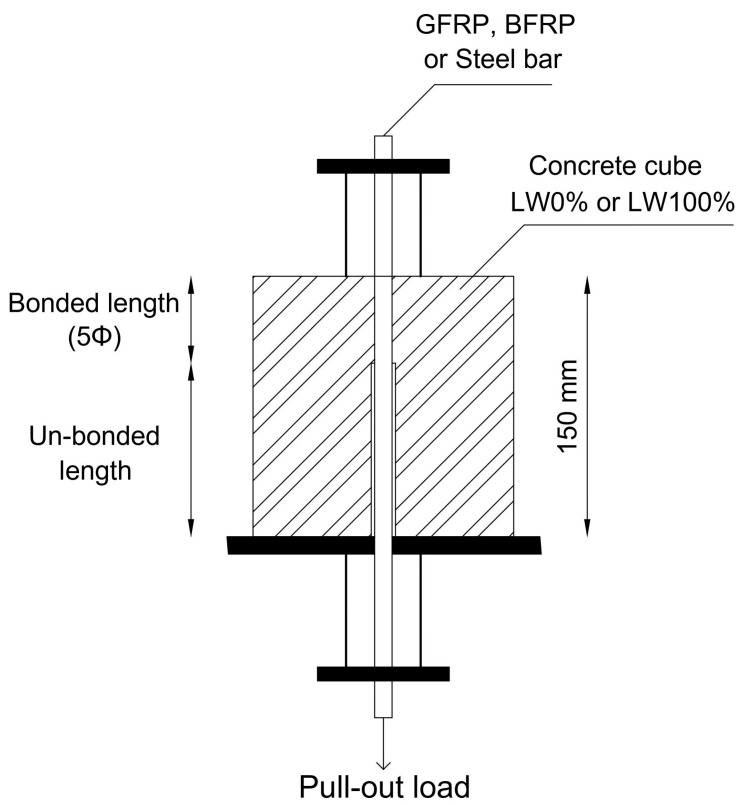
The dimensions and the test arrangement.

For testing the bond performance of reinforcing GFRP, BFRP, and steel bars embedded in LWC, P-O tests were conducted on twelve 150 mm cubes of SCC in accordance with the RILEM recommendation for steel reinforcement [35,36]. One bar was embedded in each cube with an embedment length of five times the nominal diameter of the bars, as shown in Figure 3. For each reinforcement type, four specimens were prepared: two from concrete LW0%, and two from concrete LW100%. The adopted nomenclature for the cubes was the following: St-LW0% and St-LW100% for the steel-reinforced cubes with 0% and 100% of LWA, respectively; Gl-LW0% and Gl-LW100% for the GFRP cubes with 0% and 100% of LWA, respectively; and finally Ba-LW0% and Ba-LW100% for the BFRP cubes with 0% and 100% of LWA, respectively. In this research, displacement-controlled loading was applied by a Zwick/Roell Z400-type device with a constant loading rate of 0.05 mm/s. The loading force was increased up to the failure of the bond or the splitting of the concrete cube. Figure 3 illustrates the specimen and test arrangement.

### 2.3. Computational Modeling

After discussing and analyzing the results from the experimental side in the previous section, an inverse analysis was executed by ATENA 3D. ATENA 3D applies the FE method for nonlinear structural analysis [26]. Six P-O model were prepared using the software: St-LW0%, St-LW100%, Gl-LW0%, Gl-LW100%, Ba-LW0%, and Ba-LW100%. The compressive strength of the concrete was entered into the software. Regarding the reinforcing bars, the properties and bond stress–slip curves were defined according to the experimental P-O test results [37].

**Figure 4:**
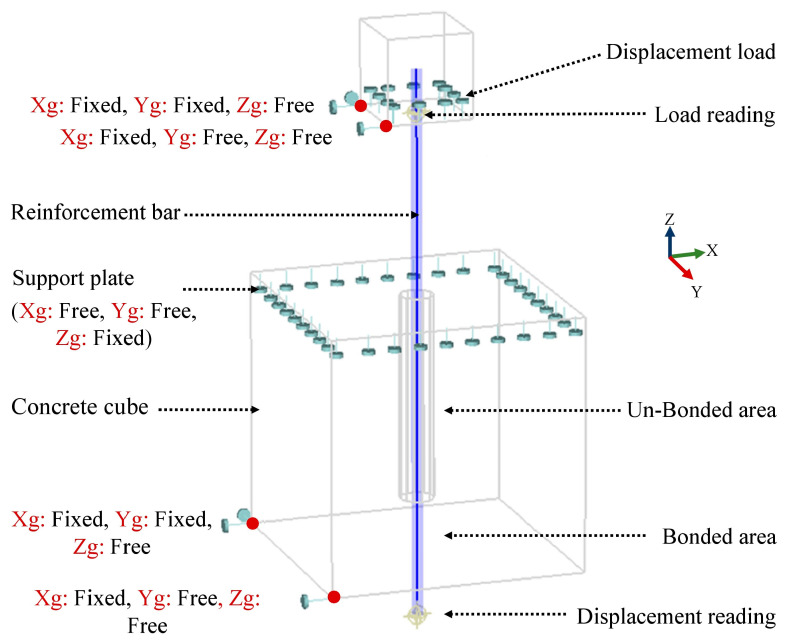
FE model of the pull-out test.

The constitutive models of each material were described in Cervenka [26]. In the ATENA 3D software, there is no option to apply a force to the reinforcement. The manual recommends defining a small cube—at the gripping end of the reinforcing bar—with a sufficiently large Young’s modulus compared to that of the reinforcing bar and applying the displacement load to this cube. The deformation of the elastic cube was negligible due to its small value compared to the other deformations [38]. For supports, the top cube was supported at two joints with the first joint supported in the one direction in the horizontal plane and the second joint in the lateral direction. As for the bottom cube, it was supported in the same way as well, and the top surface was supported in the vertical direction. Figure 4 explains in detail the numerical scheme used in this research. The loading history for the analysis was defined, which consisted of load steps, and each load step was defined as a combination of load cases. For the solution method, Newton–Raphson was used in the software. Detailed information of the model used is provided in Supplementary Materials in Tables S1–S4.

The objective of defining the loading history was to keep increasing the load up to failure. The Newton–Raphson method uses the concept of strength increments, which are calculated iteratively for each step loading until the differential function variables converge. For each new iteration of a load step, the force was kept constant, and the displacement was redefined until the tangent line of the force increment found the force versus deflection curve. During nonlinear analysis, it is useful to monitor forces and displacements in the model. The monitored data can provide important information about the state of the model and obtain the required bond stress–slip curve. Therefore, two monitoring points were defined in the numerical modeling. The first point monitored the force applied to the reinforcing bar. The second monitored the displacement at the free end of the reinforcing bar.

## 3. Results

### 3.1. Fresh Properties

**Figure 5:**
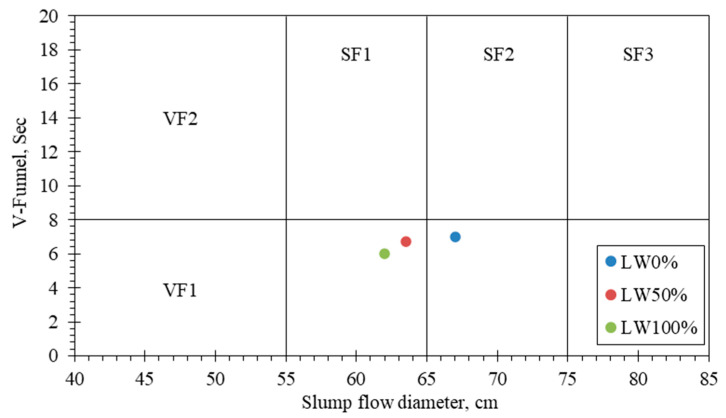
Processing window for SCC produced by LWA.

Figure 5 shows the relationship (processing window) between V-funnel flow time and slump flow diameter for all mixes. It shows the same range of European guidelines for SCC that are specified for normal-weight aggregate. Thus, European guidelines for SCC that are proposed to avoid segregations or stagnations are suitable for SCC produced by LWA. Flow time increased by increasing LWA dosage, while flow diameter decreased, which was due to the higher porosity of LWA compared to the normal weight aggregate. A higher dosage of chemical admixtures was required for the SCC produced by LWA to manage the workability of the mixes. It is worth noting that the SCC produced by 50% or 100% of LWA was classified as the VS1 viscosity class with an SF1/SF2 slump flow diameter, which can be applicable for constructing ramps and walls/columns.

### 3.2. Mechanical Properties

**Figure 6:**
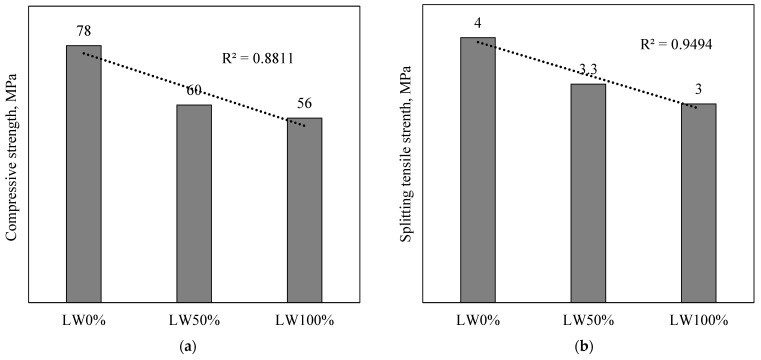

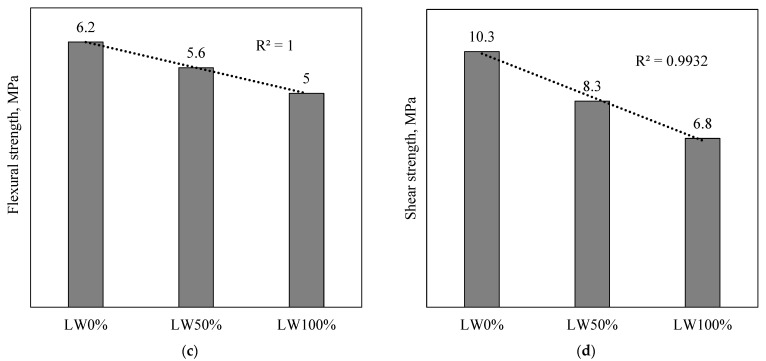
Reduction of mechanical strength of SCC by increasing the LWA proportion: (**a**) compressive strength, (**b**) splitting tensile strength, (**c**) flexural strength, and (**d**) shear strength.

The average mechanical properties, namely (a) compressive strength, (b) splitting tensile strength, (c) flexural strength, and (d) shear strength of SCC produced with different proportions of LWA, are presented in Figure 6. It was observed that increasing the ratio proportions of LWA caused a linear reduction in the mechanical properties of SCC with a high coefficient of determination. The average compressive strength of LW100% decreased by 28% compared to that of LW0% (control mix); this behavior corresponds to Maghsoudi et al. [39]. The reduction of compressive strength and mechanical properties in general by increasing the LWA proportion is due to several reasons, including the following:

1. Expanded clay has high porosity that increases the water absorption of concrete,
2. Increasing the pore area and thickness of the interfacial zone in LWC renders cement particles unable to bind intimately with the expanded clay,
3. Expanded clay has a low density and crushing strength, which leads to a reduction in the strength of SCC.

The splitting tensile strength of LWC is associated with its compressive strength and depends significantly on the curing condition [40]. Figure 6b shows that the splitting tensile strength decreases from 4 to 3.3 MPa when 50% of the coarse natural aggregate is replaced with LWA, and the reduction is higher when higher substitution is conducted. In the case of flexural strength, producing SCC by 100% of LWA decreased the flexural strength by 19% compared to that of LW0% (control mix). Other researchers [41] have observed the same behavior in their study and concluded that producing SCC with 10 mm expanded clay size gave the best flexural strengths. Direct shear test results showed a reduction by increasing the LWA dosage, which was due to the potentially brittle nature of LWC, where LWC is more brittle than that of normal weight concrete [42]. The direct shear strength of the concrete was observed by Nogueira [43] to be approximately 1.5 times the tensile strength. Shear strength for LW0%, LW50%, and LW100% were 10.3 MPa, 8.3 MPa, and 6.8 MPa, respectively.

### 3.3. Schmidt Hammer Rebound Value and Density

**Figure 7:**
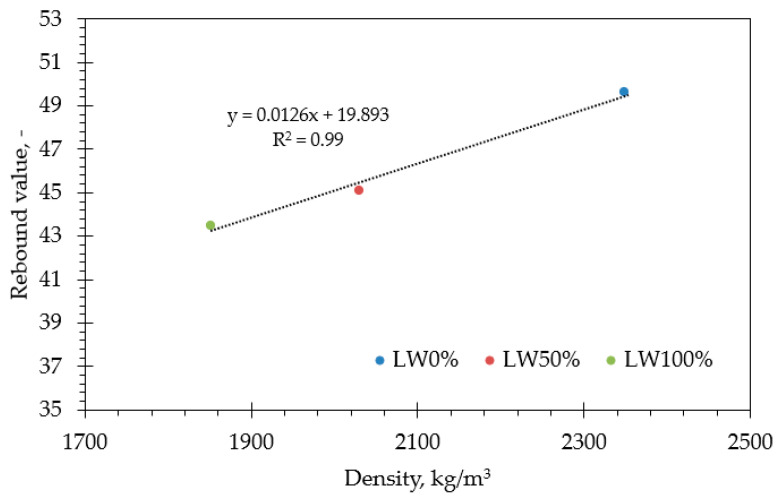
Correlation between rebound values and density of SCC produced with different LWA proportions.

The Schmidt hammer test is a non-destructive test used to measure the elastic properties or the concrete strength. Rebound values and density measurements were carried out on the SCC concrete cubes with different LWA proportion ratios. They were correlated linearly with a coefficient of determination of 0.99, as shown in Figure 7, as both of them were affected by the aggregate type [44]. Rebound values were influenced by LWA’s lower strength and bulk density as well as its high absorptivity. Rebound values decreased from 49.64 for LW0% (red circle) to 43.47 for LW100% (green circle), while the density of LW100% decreased by 21.2% compared to that of LW0%.

### 3.4. Pull-Out Test

#### 3.4.1. Experimental Evaluation

The results of P-O tests are summarized in Table 5, including local bond strength, loaded end slip, as well as free-end slip values (at maximum bond stress). The bond strength (τ_b,max_) is defined considering uniform bond stress distribution along the bond length, calculated by dividing the load by the shear surface (Equation (1)).

(1) $$\tau_{b,\max}=\frac{F_{\mathrm{ult}}}{\pi \;\cdot \;Ø\;\cdot {\;l}_{b}}$$

where F_ult_—maximum load (N); Ø—nominal bar diameter (mm); l_b_—bond length (equal to 5Ø).

#### 3.4.2. Bond Failure Modes

The possible failure modes in the case of P-O tests are splitting of the concrete block, P-O of bars, or fracture of bars. To obtain meaningful information about the bond behavior, failure should happen by P-O of the bars. Keeping the diameter of the bars relatively low, as well as the bond length equal to 5Ø, was enough to achieve the desired failure type during all the experiments.

#### 3.4.3. Bond Stress–Slip Relationships

**Figure 8:**
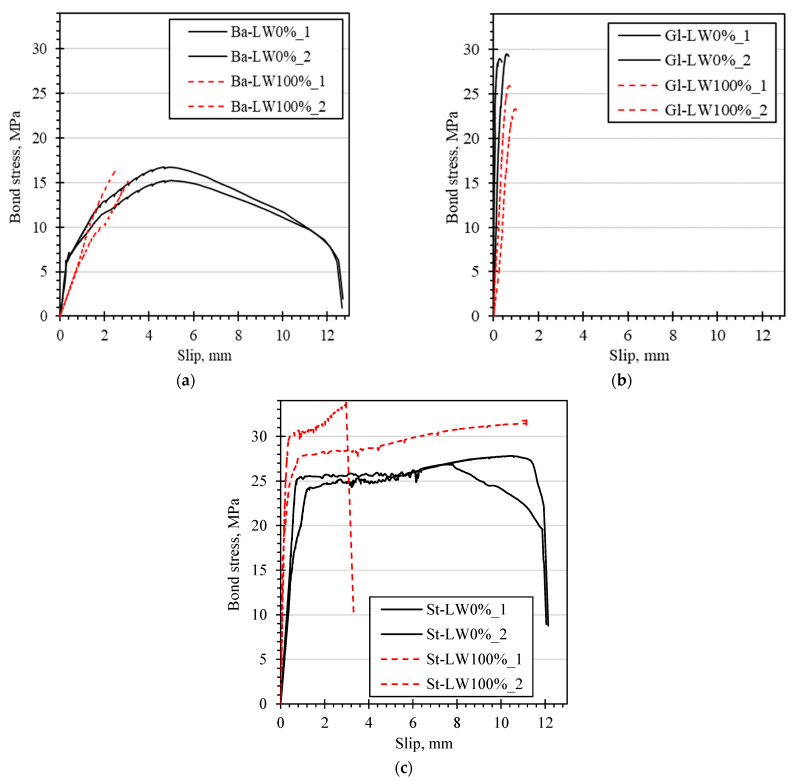
Bond stress–slip (loaded end) diagrams of different bars: (**a**) BFRP with a helically wrapped surface, (**b**) GFRP with a helically wrapped and sand coated surface, (**c**) steel ribbed surface.

Bond stress–slip relationships provide insights into the bond behaviors between reinforcements and concrete (Figure 8). It could be observed that during the P-O tests, bond failure of GFRP bars with helically wrapped and sand coated surfaces always happened in a brittle manner (no descending part of the diagram), while other types of bars failed in a ductile mode, allowing the bond strength to gradually decrease after the bond strength (maximum bond stress) was reached. The highest slip value recorded at the peak bond stress was observed for BFRP bars with a helically wrapped surface (in the case of steel bars, the observed higher slip values were only because the bars were already yielding).

The curves show that LWC specimens—in the case of GFRP and BFRP bars—had lower bond stiffness, and the slope of the ascending branch was lower. This made the bond behavior more ductile, which however might be disadvantageous for serviceability limit state design.

In general, the bond stress–slip responses were characterized by the linear part of the ascending branch when the bond surface was still not totally damaged, followed by a nonlinear section up to peak bond stress (bond strength) due to the accumulated damage on the bar surface and in the concrete surrounding the bar. The post-peak phase (descending branch) varied depending on the bar type. However, in the case of HWSC bars, no descending part was visible due to the sudden shearing of the surface of the FRP bar.

**Figure 9:**
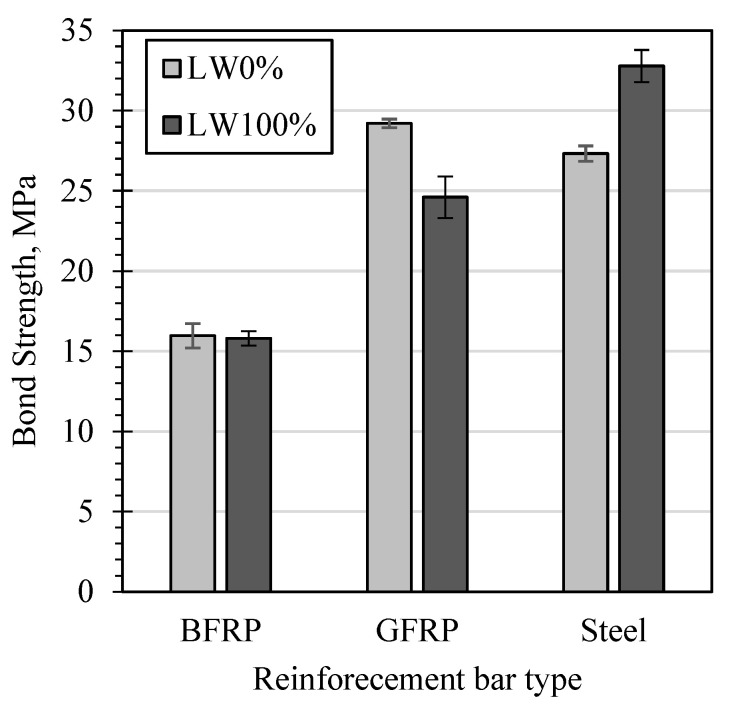
Comparing the bond stress–slip curves between the experiment and numerical modeling.

It can be observed in Figure 9 that the bond strength of BFRP bars was practically not affected by the replacement of coarse aggregate with LWA, whilst the GFRP bar showed lower bond strength values of LWC specimens. Contrarily, in the case of steel bars, both the bond strength and bond stiffness were higher for LWC specimens than for those of normal concrete.

### 3.5. Numerical Evaluation

**Figure 10:**
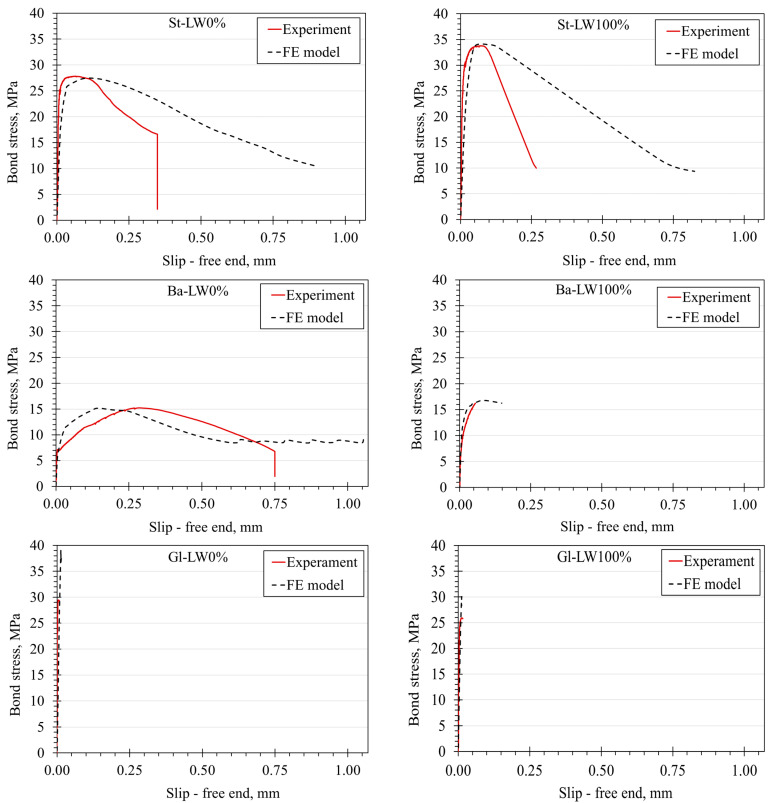
Comparison of the bond stress–relative slip curves between the experiment and numerical modeling.

The primary purpose of modeling the P-O tests was to plot and validate the bond stress–slip curves for St-LW0%, St-LW100%, Gl-LW0%, Gl-LW100%, Ba-LW0%, and Ba-LW100% with the experimental as well as presenting the crack width and pattern of the concrete cube. Due to the matching of most of the results of the two specimens for each case, and for simplicity of the comparison between the experimental and FE results, one curve for each case was plotted. Figure 10 shows the comparison of the experimental and numerical results for each case. The numerical results of the steel bar, BFRP, and GFRP embedded in LW0% and LW100% mixes agreed reasonably well with the experimental results. The same results were found in the previous studies by Tavares et al. [28] and Mesbah et al. [45]. The nonlinear FE analyses showed good agreements on the overall bond stress–slip curve, which expressed the real behavior.

**Figure 11:**
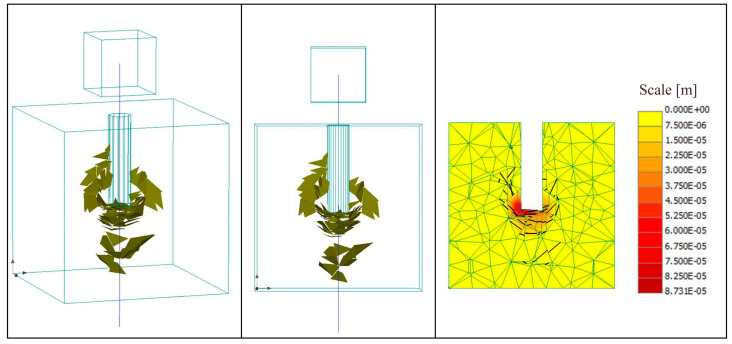
Crack width and pattern of the concrete cube after the P-O failure of Ba-LW0%.

ATENA 3D software can be effectively used to support and extend experimental investigations for innovative solutions in the field of connections between reinforcing bars and concrete. Figure 11 illustrates the crack width and pattern of the concrete cube after the P-O failure of Ba-LW0%. The cracks occurred inside the specimen around the bond area where the bond between the reinforcing bar and the concrete hindered the P-O process; no obvious cracks were found at the surface of the concrete cube. This confirms that the failure type was a P-O failure. It shows the visible cracks in reality, where the very small cracks were filtered out. Regarding the maximum value of the bond stress (bond strength) and the corresponding slip value, the values were compared between the laboratory experiment and the numerical modeling and are presented in Table 6.

## 4. Conclusions

This study was conducted to evaluate the properties of SCC produced by expanded clay LWA as well as its bond behavior when steel, BFRP, or GFRP reinforcement bars were used. The provided comparison was based on performed experimental tests as well as by computational modeling. The bond behavior was evaluated experimentally by using the P-O test type, and then the ATENA 3D was developed to describe the experimental results numerically. The results showed that SCC can be produced with LWA; however, it somehow linearly loses its fresh and mechanical performances by increasing the LWA replacement dose. The loss of its mechanical performance did not exceed 28%, and that was in the case of using 100% replacement of coarse aggregate by LWA. The bond strength of BFRP bars was not affected by the replacement of coarse aggregate by LWA, whilst the GFRP bars showed lower bond strength values of LWC specimens. Contrarily, in the case of steel bars, both the bond strength and bond stiffness were higher for LWC specimens than for those of normal weight concrete. Bond behavior in the case of GFRP bar- and BFRP bar-reinforced specimens showed more ductile behavior compared to those that were reinforced with steel bars. The numerical results of the P-O test agreed reasonably well with the experimental results. ATENA 3D software showed that it can be effectively used to support and extend experimental investigations for innovative solutions in the field of connections between reinforcing bars and concrete, and that it can be successfully used in follow-up research.

## Figures and Tables

**Table 1:** SCC mixing proportions.

Mix	Proportions in kg/m^3^						
	Cement	Fine Aggregate	Coarse Aggregate		Glenium C300	Glenium 51	Water
		Natural Sand	Normal Weight Aggregate	LWA			
		0/4 (mm)	4/8 (mm)				
**LW0%**	500	785	960	-	0.75	0.75	175
**LW50%**	500	785	480	232	1.50	2.00	175
**LW100%**	500	785	-	464	2.25	3.75	175

**Table 2:** Properties of LWA.

Parameter	Expanded Clay	
Oven-dry density (kg/m^3^)	2620	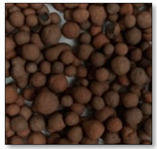
Bulk density (kg/m^3^)	359	
Particle density (kg/m^3^)	650	
Water absorption (%)	18.3	
Particle porosity (%)	75.2	
Crushing resistance (MPa)	2	

**Table 3:** Properties of reinforcement bars.

Property	Steel B500B	GFRP	BFRP
Tensile strength (MPa)	540	920	1100
Yield strength (MPa)	500	-	-
Modulus of elasticity (MPa)	200,000	55,500	70,000
Ultimate strain (%)	5	1.68	2.2
Density (kg/m^3^)	7850	2100	1900
Nominal diameter (mm)	8	10	14

**Table 4:** Classes of consistency and viscosity of SCC.

**Consistence Classes Expressed by Slump-Flow**	
**SF1**	When slump flow is 550 to 650 mm
**SF2**	When slump flow is 660 to 750 mm
**SF3**	When slump flow is 760 to 850 mm
**Viscosity Classes Expressed by V-Funnel Time**	
**VF1**	When V-funnel time is ≤8 s
**VF2**	When V-funnel time is 9 to 25 s

**Table 5:** P-O test results of different reinforcement bars.

Concrete Symbol	Fiber Type	Surface Type	Nominal Diameter	Test No.			Bond Strength		Loaded End Slip		Free End Slip	
					F_ult_	F_ult_ ^a^	τ_b,max_	τ ^a^_b,max_	s_le,τb,max_	s ^a^_le,τb,max_	s_le,τb,max_	s ^a^_fe,τb,max_
			mm		kN	kN	MPa	MPa	mm	mm	mm	mm
LW0%	BFRP	HW	14	1	51.48	49.15	16.73	15.97	4.677	4.827	0.279	0.282
				2	46.83		15.22		4.977		0.285	
	GFRP	HWSC	10	1	46.27	45.85	29.47	29.21	0.565	0.416	0.005	0.005
				2	45.44		28.94		0.267		NA	
	Steel	Ribbed	8	1	27.94	27.45	27.80	27.32	10.346	8.919	0.060	0.032
				2	26.97		26.84		7.493		0.003	
LW100%	BFRP	HW	14	1	49.98	48.63	16.24	15.80	2.467	2.79	0.056	0.06
				2	47.28		15.36		3.109		0.055	
	GFRP	HWSC	10	1	36.59	38.63	23.30	24.60	0.961	0.82	0.011	0.01
				2	40.67		25.90		0.688		0.012	
	Steel	Ribbed	8	1	31.94	32.94	31.78	32.79	11.150	7.06	0.765	0.42
				2	33.95		33.79		2.970		0.069	

^a^—average.

**Table 6:** Comparison of the maximum bond stress and the relative slip curves between the laboratory experiment and the numerical modeling.

		LW0%		LW100%	
		Bond Strength	Slip at the Free End	Bond Strength	Slip at the Free End
		MPa	mm	MPa	Mm
Steel bar	Experiment	27.803	0.060	33.788	0.069
	FE model	27.448	0.100	34.136	0.075
BFRP	Experiment	15.217	0.285	16.241	0.056
	FE model	15.189	0.152	16.811	0.083
GFRP	Experiment	29.470	0.005	25.902	0.011
	FE model	39.130	0.013	30.010	0.012

## Data Availability

Not applicable.

## Supplementary Materials

The following supporting information can be downloaded at: https://www.mdpi.com/article/10.3390/ma15103555/s1, Table S1: Summary of material model and material properties of concrete used in the model; Table S2: Summary of material model and material properties of embedded reinforcement bar; Table S3: Material properties of bond; Table S4: Solution parameters in FEM model.

## References

[1] De Brito J., Kurda R. (2021). The past and future of sustainable concrete: A critical review and new strategies on cement-based materials. J. Clean. Prod..

[2] Li Q., Zhang L.Y., Zhang L.M., Jha S. (2021). Exploring multi-level motivations towards green design practices: A system dynamics approach. Sustain. Cities Soc..

[3] Barroqueiro T., da Silva P.R., de Brito J. (2020). High-Performance Self-Compacting Concrete with Recycled Aggregates from the Precast Industry: Durability Assessment. Buildings.

[4] Abed M.A., Tayeh B.A., Abu Bakar B.H., Nemes R. (2021). Two-Year Non-Destructive Evaluation of Eco-Efficient Concrete at Ambient Temperature and after Freeze-Thaw Cycles. Sustainability.

[5] Faraj R.H., Ali H.F.H., Sherwani A.F.H., Hassan B.R., Karim H. (2020). Use of recycled plastic in self-compacting concrete: A comprehensive review on fresh and mechanical properties. J. Build. Eng..

[6] Solyom S., Di Benedetti M., Balázs G.L. (2021). Bond of FRP bars in air-entrained concrete: Experimental and statistical study. Constr. Build. Mater..

[7] Solyom S., Balázs G.L. (2021). Analytical and statistical study of the bond of FRP bars with different surface characteristics. Compos. Struct..

[8] Abed M.A., Alkurdi Z., Kheshfeh A., Kovács T., Nehme S. (2021). Numerical Evaluation of Bond Behavior of Ribbed Steel Bars or Seven-Wire Strands Embedded in Lightweight Concrete. Period. Polytech. Civ. Eng..

[9] Alkhrdaji T., Fyfe E.R., Karbhari V.M., Schupack M., Bakis C.E., Ganjehlou A., Korff J.G., Scott D.W., Balaguru P.N., Gee D.J. (2004). ACI440.3R—Guide Test Methods for Fiber-Reinforced Polymers (FRPs) for Reinforcing or Strengthening Concrete Structures.

[10] Sólyom S., Balázs G.L., Di Benedetti M., Guadagnini M., Zappa E. Bond Strength of GFRP Rebars in Concrete at Elevated Temperature. Proceedings of the Advanced Composites in Construction.

[11] Mousavi S.S., Dehestani M., Mousavi K.K. (2017). Bond strength and development length of steel bar in unconfined self-consolidating concrete. Eng. Struct..

[12] Ding X.X., Geng H.B., Zhao M.L., Chen Z., Li J. (2021). Synergistic Bond Properties of Different Deformed Steel Fibers Embedded in Mortars Wet-Sieved from Self-Compacting SFRC. Appl. Sci..

[13] Castel A., Vidal T., Francois R. (2010). Bond and cracking properties of self-consolidating concrete. Constr. Build. Mater..

[14] Trad A., Ghanem H., Ismail R. Bond Behaviour of Structural Lightweight Concrete. High Tech Concrete: Where Technology and Engineering Meet. Proceedings of the 2017 fib Symposium.

[15] Bogas J.A., Gomes M.G., Real S. (2014). Bonding of steel reinforcement in structural expanded clay lightweight aggregate concrete: The influence of failure mechanism and concrete composition. Constr. Build. Mater..

[16] Gao D.Y., Yan H.H., Fang D., Yang L. (2020). Bond strength and prediction model for deformed bar embedded in hybrid fiber reinforced recycled aggregate concrete. Constr. Build. Mater..

[17] Larsen I.L., Thorstensen R.T. (2020). The influence of steel fibres on compressive and tensile strength of ultra high performance concrete: A review. Constr. Build. Mater..

[18] Qi A., Liu X.H., Xu R.J., Huang Y.S. (2020). Bond behavior of steel reinforcement in concrete containing ferronickel slag and blast furnace slag powder. Constr. Build. Mater..

[19] Wang J., Su H., Du J.S. (2020). Corrosion Characteristics of Steel Bars Embedded in Recycled Concrete Beams under Static Loads. J. Mater. Civ. Eng..

[20] Han S.W., Zhou A., Ou J.P. (2021). Relationships between interfacial behavior and flexural performance of hybrid steel-FRP composite bars reinforced seawater sea-sand concrete beams. Compos. Struct..

[21] Zamora-Castro S.A., Salgado-Estrada R., Sandoval-Herazo L.C., Melendez-Armenta R.A., Manzano-Huerta E., Yelmi-Carrillo E., Herrera-May A.L. (2021). Sustainable Development of Concrete through Aggregates and Innovative Materials: A Review. Appl. Sci..

[22] Cadenazzi T., Dotelli G., Rossini M., Nolan S., Nanni A. (2020). Cost and environmental analyses of reinforcement alternatives for a concrete bridge. Struct. Infrastruct. Eng..

[23] Huang Z.J., Chen W.S., Tran T.T., Pham T.M., Hao H., Chen Z.Y., Elchalakani M. (2021). Experimental and numerical study on concrete beams reinforced with Basalt FRP bars under static and impact loads. Compos. Struct..

[24] Alkurdi Z. Influence of Concrete Compressive Strength on Transfer Length in Pretensioned Concrete Members Using 3D Nonlinear FEM Analysis. Proceedings of the 6th International Conference on Civil, Structural and Transportation Engineering (ICCSTE’21).

[25] Smith M. (2009). ABAQUS/Standard User’s Manual.

[26] Cervenka V., Jendele L., Cervenka J. (2007). Atena Program Documentation Part 1 Theory.

[27] Yu H., Jeong D. Finite Element Bond Modeling for Indented Wires in Pretensioned Concrete Crossties. Proceedings of the 2016 Joint Rail Conference.

[28] Tavares A.J., Barbosa M.P., Bittencourt T.N., Lorrain M. (2014). Bond steel-concrete: Simulation analysis of the pull-out tests and APULOT using the program ATENA. Rev. IBRACON Estrut. Mater..

[29] Cheung A.K., Leung C.K., Kabele P. Finite element study on bond behavior of steel bar and HSCC/HSFRCC. Proceedings of the 7th International Conference on Fracture Mechanics of Concrete and Concrete Structures.

[30] EFNARC (2005). The European Guidelines for Self-Compacting Concrete, Eur. Guidellins Self Compacting Concrete 63. http://www.efnarc.org/pdf/SCCGuidelinesMay2005.pdf.

[31] (2009). Testing Hardened Concrete. Compressive Strength of Test Specimens.

[32] (2004). Bituminous Mixtures. Test Methods for Hot Mix Asphalt. Part 23: Determination of the Indirect Tensile Strength of Bituminous Specimens.

[33] (2009). Testing Hardened Concrete. Flexural Strength of Test Specimens.

[34] Abed M.A., Fořt J., Naoulo A., Essa A. (2021). Influence of Polypropylene and Steel Fibers on the Performance and Crack Repair of Self-Compacting Concrete. Materials.

[35] Windisch A. (1985). A modified pull-out test and new evaluation methods for a more real local bond-slip relationship. Mater. Struct..

[36] RILEM (1994). RC 6 Bond Test for Reinforcement Steel. 2. Pull-Out Test, 1983.

[37] Cervenka J., Prochazkova Z., Sajdlova T. (2017). ATENA Program Documentation Part 11 Troubleshooting Manual.

[38] Cervenka J., Prochazkova Z., Sajdlova T. (2017). ATENA Program Documentation Part 4-2 Tutorial for Program ATENA 3D.

[39] Maghsoudi A.A., Mohamadpour S., Maghsoudi M. (2011). Mix design and mechanical properties of self compacting light weight concrete. Int. J. Civ. Eng..

[40] Bogas J.A., Nogueira R. (2014). Tensile strength of structural expanded clay lightweight concrete subjected to different curing conditions. KSCE J. Civ. Eng..

[41] Nahhab A.H., Ketab A.K. (2020). Influence of content and maximum size of light expanded clay aggregate on the fresh, strength, and durability properties of self-compacting lightweight concrete reinforced with micro steel fibres. Constr. Build. Mater..

[42] Carrillo J., Lizarazo J.M., Bonett R. (2015). Effect of lightweight and low-strength concrete on seismic performance of thin lightly-reinforced shear walls. Eng. Struct..

[43] Nogueira C.L. (2020). Anti-plane shear strength of plain concrete. Mater. Today Commun..

[44] Aydin F., Saribiyik M. (2010). Correlation between Schmidt Hammer and destructive compressions testing for concretes in existing buildings. Sci. Res. Essays.

[45] Mesbah H.A., Benzaid R., Benmokrane B. (2017). Evaluation of bond strength of FRP reinforcing rods in concrete and FE modelling. Int. J. Civ. Eng. Constr. Sci..

