# Supplementary material for: Bond Behavior of FRP Bars in Lightweight SCC under Direct Pull-Out Conditions: Experimental and Numerical Investigation
Source: Materials (Basel). 2022 May 16;15(10):3555. doi: 10.3390/ma15103555 (PMC9145959; doi:10.3390/ma15103555)
Supplement: Supplementary file 1 [file materials-15-03555-s001.zip › materials-1702376-supplementary.pdf]

Supplementary Materials

Main input data

Table S1: Summary of material model and material properties of concrete used in the model.

	LW0%	LW100%
Material model	3D Nonlinear Cementitious 2 Model	
Elastic Modulus	42.31 GPa	38.4 MPa
Poisson's ratio	0.2	0.2
Tensile strength	4 MPa	3 MPa
Compressive strength (cube)	78 MPa	56 MPa
Compressive strength (cylinder)	66.3 MPa	47.6 MPa
Specific Fracture Energy (G_f)	109.5 N/m	87.82 N/m
Critical compressive displacement (w_d)	-5x10^{-4} m	-5x10^{-4} m
Plastic strain at compressive strength ε_{cp}	-1.567x10^{-3} m	-1.239x10^{-3} m
Type of tension softening	Exponential	
Crack Model	Fixed	

Table S2: Summary of material model and material properties of embedded reinforcement bar.

	Steel B500B	GFRP	BFRP
Material model		Reinforcement	
Material type	Bilinear	Linear	Linear
Elastic Modulus	200 GPa	55.5 GPa	70 GPa
Yield strength σy	500		
Hardening	Linear		

Table S3: Material properties of bond.

Material type	Bond for reinforcement
Function	Multi-linear

Table S4: Solution parameters in FEM model.

Solution Method	Newton-Raphson
Stiffness/update	Tangent/each iteration
Number of iterations	30
Error tolerance	0.01
Line search	On, with iterations
